# Multifunctional bioactivity of eco-friendly *Penicillium gladioli* extract against *Toxoplasma gondii* and *Pseudomonas aeruginosa*

**DOI:** 10.1038/s41598-025-23921-z

**Published:** 2025-11-26

**Authors:** Engy Elekhnawy, Ehssan Moglad, Nizar Sirag, Rehab Ahmed, Noha Abd El-Salam, Salwa S. Younis, Hoda A. Rashed, Eman A. Elmorsy, Lamiaa A. Salama, Omnia Momtaz Al-Fakhrany

**Affiliations:** 1 Microbiology and Immunology Department, Faculty of Pharmacy, Tanta University, Tanta, 31527 Egypt; 2 Department of Pharmaceutics, College of Pharmacy, Prince Sattam bin Abdulaziz University, P.O. Box 173, 11942 Alkharj, Saudi Arabia; 3 Division of Pharmacognosy, Department of Natural Products and Alternative Medicine, Faculty of Pharmacy, University of Tabuk, Tabuk 71491, Saudi Arabia; 4 Division of Microbiology, Immunology and Biotechnology, Department of Natural Products and Alternative Medicine, Faculty of Pharmacy, University of Tabuk, 71491 Tabuk, Saudi Arabia; 5 Research center and Measurements, Tanta university, Tanta, Egypt; 6 Medical Parasitology, Faculty of Medicine, Alexandria University, Alexandria, Egypt; 7 Microbiology and Immunology Department, Faculty of Pharmacy, Horus University, New Damietta, 34518 Egypt

**Keywords:** Tachyzoites, Biofilm, *Pseudomonas aeruginosa*, GC-MS, Soil, Biochemistry, Biotechnology, Microbiology

## Abstract

The soil fungus *Penicillium gladioli* was identified by sequencing of the internal transcribed spacer (ITS) region. Gas chromatography-mass spectroscopy of the extract showed 50 compounds. Among the detected peaks, n-hexadecanoic acid showed the largest relative peak area (7.989% of the total ion chromatogram), followed by phenol, 2-methyl-5-(1-methylethyl) (6.543%). Regarding the in vivo anti-toxoplasma potential, there was an enhancement of the histological features of the liver of Swiss albino mice with a substantial decrease (*p* < 0.05) in the inflammatory mediators, including cyclooxygenase-2, tumor necrosis factor-alpha, interleukin-6, and interleukin-1β. The colorimetric determination of the nitric oxide and malondialdehyde in the liver of the fungal extract-treated group revealed its antioxidant effect by significantly reducing (*p* < 0.05) the oxidative stress markers. *P. gladioli* extract established antibacterial potential on *P. aeruginosa* bacteria with a minimum inhibitory concentration of 64 to 512 µg/ml. Moreover, it demonstrated antibiofilm potential using crystal violet assay and SEM. Also, 45% of the isolates displayed downregulation of the *lasR*, *lecA*, and *pelA* biofilm genes.

**Supplementary Information:**

The online version contains supplementary material available at 10.1038/s41598-025-23921-z.

## Introduction

Diverse microorganisms are present in the soil, and they can adapt to environmental conditions by yielding various natural substances or metabolites to deal with harsh habitat characteristics^1^. Owing to their great pharmacological activities, such metabolites could be employed as pharmaceutical products. Fungi constitute a large proportion of soil microbes and can produce many bioactive compounds. Therefore, saprophytic fungi are a sustainable source of various compounds with different biological activities^1^.

*Toxoplasma gondii* is an a parasite that causes toxoplasmosis, an ailment affecting millions of people^2^. Usually, acute toxoplasmosis is followed by asymptomatic latent infection, and *T. gondii* encysts in several organs. Such latent infections often reactivate in the immunocompromised hosts, triggering severe fatal illness if left untreated^3^.

Owing to available treatment options still hampered by severe adverse effects, there is a strong push to develop other new options of therapy for toxoplasmosis. Consequently, there is a crucial necessity to find innovative anti-toxoplasma drugs or to improve the existing ones^4,5^.

*Pseudomonas aeruginosa* is a pathogenic bacterium with various virulence factors, like biofilm formation. Biofilm is a group of bacterial cells in an extracellular matrix. The biofilm formation facilitates the communication between bacterial cells and resistance gene transfer. In addition to the multiple virulence factors of *P. aeruginosa*, it can resist multiple traditional antibiotics. The recent rising antibiotic resistance rates among bacteria have elicited a great demand to seek alternative chemicals from natural origin to overcome drug-resistant microbes^6^.

Soil is a suitable environment for the growth of various microbes. So, it is highly explored to elucidate microorganisms that have the capability to yield valuable bioactive products, like antibacterial, antifungal, and antiparasitic agents^7^.

Thus, much research was performed to evaluate the potential antibacterial and antivirulence action of different natural substances^8^. Saprophytic fungi like *Aspergillus niger*,* Fusarium oxysporum*,* and Emericella nidulans*^9^ could produce various secondary metabolites that possess antibacterial action.

**Fig. 1:**
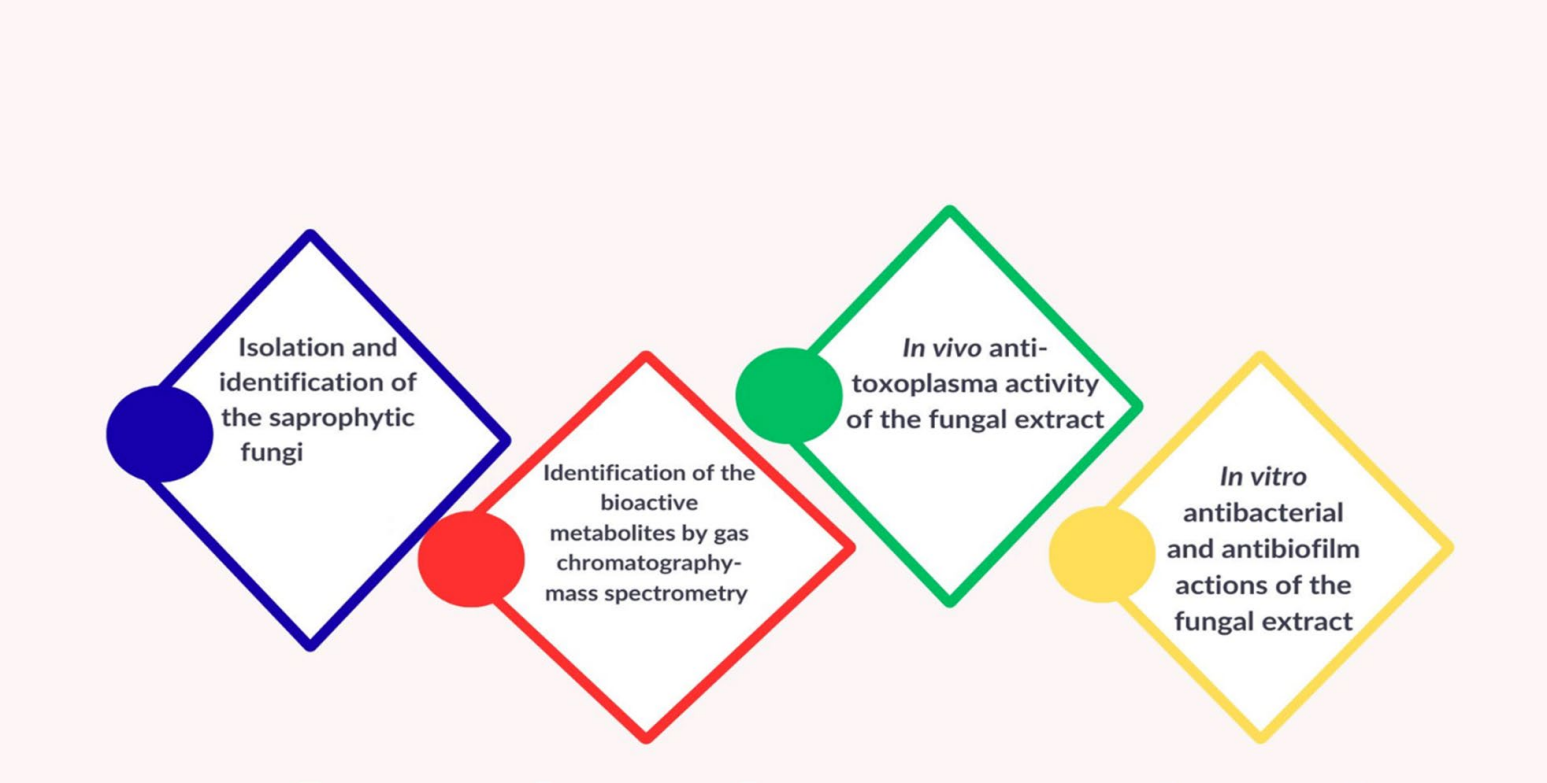
An illustrative diagram for the framework of the current study.

From this point of view, the anti-toxoplasma and antibacterial effects of the saprophytic fungi *Penicillium gladioli* extract were investigated in infected mice with virulent RH HXGPRT(−) strain of *T. gondii* and *P. aeruginosa* clinical isolates, respectively. An illustrative representation of the current investigation’s framework is shown in Fig. 1.

## Materials and methods

### Isolation of fungi from soil samples

Five grams of different samples of soil were gathered in sterile polythene bags from Tanta, Egypt, at various locations. In the microbiology laboratory, the soil samples were diluted in sterile water (1 g of soil in 10 mL of water). Then, the prepared suspension was added to potato dextrose agar (PDA, Oxoid, USA) plates, which were incubated at 28 °C for 1 week. Then, isolation of the pure fungal culture was performed on PDA plates^10^.

### Identification of the saprophytic fungi

The isolated saprophytic fungus was obtained as a pure culture on PDA plates^11^ to be recognized by sequencing of the internal transcribed spacer (ITS) region^12^. The sequence of the utilized primer was 5′-TCCGTAGGTGAACCTGCGG-3′ in the forward direction and 5′-TCCTCCGCT TATTGATATGC-3′ in the reverse direction. The sequences of the amplified products were determined at Macrogen Co., Korea. Then, the resulting sequences were deposited in the Gene Bank (https://blast.ncbi.nlm.nih.gov/Blast.cgi) under the accession number PV893029. The BLAST analysis revealed 100% identity with *Penicillium gladioli* (accession number AF033480). Using the MEGA 7.0 program, a phylogenetic tree was constructed.

### Preparation of the fungal fermentation broth

The identified saprophytic fungal isolate was cultivated at 25 °C for 10 days in a potato dextrose liquid, and the ethyl acetate fungal extract was prepared, as previously explained^13^. The crude fermentation broth was thoroughly blended for the selected isolate (10^7^ CFU/ml) and centrifuged. Liquid supernatant was extracted with an equal amount of ethyl acetate and repeated three times. The organic solvent extract was then evaporated under reduced pressure to yield the crude metabolite for screening its anti-toxoplasma and antibacterial activities.

### Identification of bioactive metabolites by gas chromatography-mass spectrometry (GC-MS)

The Perkin Elmer model (Clarus 580/560 S) was employed in the column analysis (Rxi-5Sil MS column 30 m, 0.25 mm ID, 0.25 df), employing helium as a carrier gas^14^. During the chromatographic run, the temperature of the injector was set at 280 °C. One microliter of the fungal extract was injected into the scanning instrument for 30 min, with an initial oven temperature of 60° C for 10 min, ramp of 10 °C/min to 280 °C for 6 min, split 20:1, solvent delay, 3 min. The conditions for the mass detector were transferred line temperature of 280 °C, ion source temperature of 200 °C, and the effect of ionization mode at 70 eV, a scan time of 0.2 s, and a scan interval of 0.1 s. Given fragments from 50 to 600 Da, the component spectrum was compared with the spectrum database of recognized components held in the GC-MS NIST library.

### Parasite

The virulent RH HXGPRT(−) strain of *T. gondii* was obtained from the medical parasitology department, faculty of medicine, Alexandria University, Egypt, and it was conserved by its serial passage in Swiss albino mice^15^.

### Experiment

Thirty-six Swiss albino mice (4–6 weeks old, weighing 20–25 g) were obtained from Alexandria University. The mice were retained in conditions of 23 ± 2 °C/45–55% relative humidity. Essential procedures were performed to provide proper care. The procedures were accepted by the ethics committee of the Faculty of Medicine, Alexandria University, Egypt (approval number 0306733).

The mice were randomly grouped into three groups (each composed of 12 mice).

Group I was intra-peritoneally (IP) infected with 3500 tachyzoites of *T. gondii*, and it wasn’t treated.

Group II was IP infected with 3500 tachyzoites of *T. gondii* and then treated orally with spiramycin (Medical Union Pharmaceuticals, Egypt, 200 mg/kg/day) for five days^5^.

Group III was IP infected with 3500 tachyzoites of *T. gondii* and then treated orally with *P. gladioli* fungal extract (150 mg/kg/day) for five days^16^.

On the sixth day, half of the mice from all groups were sacrificed by cervical dislocation after anaesthesia by isoflurane inhalation, and the rest were employed to calculate the survival rate^17^.

### Anti-toxoplasma effect of ***P. gladioli*fungal extract**.

The survival time and rate were estimated as previously reported^18^. The mean count of the tachyzoites was detected in one milliliter of the peritoneal fluid by a hemocytometer (HBG^®^- Germany)^19^. The parasite burden percent reduction (%R) was revealed in the experimental groups^15^ employing the formula:

$$\:\%R=\frac{\text{T}\text{a}\text{c}\text{h}\text{y}\text{z}\text{o}\text{i}\text{t}\text{e}\text{s}\:\text{m}\text{e}\text{a}\text{n}\:\text{n}\text{u}\text{m}\text{b}\text{e}\text{r}\:\left(\text{c}\text{o}\text{n}\text{t}\text{r}\text{o}\text{l}\:\text{g}\text{r}\text{o}\text{u}\text{p}\right)-\:\text{T}\text{a}\text{c}\text{h}\text{y}\text{z}\text{o}\text{i}\text{t}\text{e}\text{s}\:\text{m}\text{e}\text{a}\text{n}\:\text{n}\text{u}\text{m}\text{b}\text{e}\text{r}\:\left(\text{i}\text{n}\text{f}\text{e}\text{c}\text{t}\text{e}\text{d}\:\text{g}\text{r}\text{o}\text{u}\text{p}\right)}{\text{T}\text{a}\text{c}\text{h}\text{y}\text{z}\text{o}\text{i}\text{t}\text{e}\text{s}\:\text{m}\text{e}\text{a}\text{n}\:\text{n}\text{u}\text{m}\text{b}\text{e}\text{r}\:\left(\text{c}\text{o}\text{n}\text{t}\text{r}\text{o}\text{l}\:\text{g}\text{r}\text{o}\text{u}\text{p}\right)}\times\:100$$

### Scanning electron microscopic study (SEM)

Tachyzoites of *T. gondii* from the peritoneal exudates of mice from the different experimental groups were prepared for SEM examination, as previously reported^19^, to elucidate the consequence of the fungal extract on the parasite’s ultrastructure.

### Histopathological and immunohistochemical elucidation

As previously explained, liver from mice was preserved in 10% formalin for histopathological study^20^ after staining with hematoxylin and eosin (H&E) and periodic acid-schiff (PAS) stain^20,21^. The immunohistochemical investigations were achieved using the monoclonal antibodies for cyclooxygenase-2 (COX-2), tumor necrosis factor-alpha (TNF-α), interleukin-6 (IL-6), and interleukin-1β (IL-1β) as previously reported^22^.

### Colorimetric investigation

Colorimetric kits (Biodiagnostics, Egypt) were employed to measure the nitric oxide (NO) and malondialdehyde (MDA) in the liver at 540 nm.

### Antibacterial effect

It was illuminated by the agar well diffusion method in Muller-Hinton agar plates, employing ciprofloxacin (2 µg/mL) and DMSO, as positive and negative controls, respectively^23–25^. Twenty *P. aeruginosa* clinical isolates were tested and obtained from the pharmaceutical microbiology department, faculty of pharmacy, Tanta University. Then, after revealing the antibacterial action by measuring the diameters of the inhibition zones around the wells, the MICs were determined using 96-well microtitration plates, as formerly clarified^25^.

### Antibiofilm action

The capacity of the fungal extract to hinder the ability of *P. aeruginosa* to form biofilm was elucidated using crystal violet assay in microtitration plates^26^. Also, the consequence of the fungal extract on the biofilm gene expression (*lasR*, *lecA*, and *pelA*) was investigated using qRT-PCR. The antibiofilm action of the fungal extract was elucidated using the crystal violet assay (at 0.5 MIC values) on the biofilm formation ability of *P. aeruginosa* isolates. The values of the optical density (OD) were determined at 590 nm using ELISA reader. Then, the capability of tested isolates to form biofilm was grouped into four classes as follows: Non-biofilm former isolates (ODc < OD < 2 ODc), weak biofilm former isolates (2 ODc < OD < 4 ODc), moderate biofilm former isolates (4 ODc < OD < 6 ODc), and strong biofilm former isolates (6 ODc < OD). The cut-off OD (ODc) was identified as the product of the addition of the mean OD to three standard deviation (SD) of the negative control.

$$\:\text{O}\text{D}\text{c}=\text{m}\text{e}\text{a}\text{n}\:\text{O}\text{D}\:\text{o}\text{f}\:\text{n}\text{e}\text{g}\text{a}\text{t}\text{i}\text{v}\text{e}\:\text{c}\text{o}\text{n}\text{t}\text{r}\text{o}\text{l}+(3\times\:\text{S}\text{D}\:\text{o}\text{f}\:\text{n}\text{e}\text{g}\text{a}\text{t}\text{i}\text{v}\text{e}\:\text{c}\text{o}\text{n}\text{t}\text{r}\text{o}\text{l})$$

The effect of the fungal extract was studied on the expression levels of the biofilm genes (*lasR*, *lecA*, and *pelA*) by qRT-PCR. After growing the isolates in TSB in the presence and absence of 0.5 MICs, they were incubated overnight at 37 °C. After the incubation period, cells were harvested by centrifugation and immediately stored at − 80 °C. The total RNA from *P. aeruginosa* isolates was extracted and purified using TRIzol^®^ reagent (Life Technologies, USA) following the manufacturer protocol. Reverse transcription was employed using QuantiTect Reverse Transcription kit (Qiagen, Germany). After that, the formed cDNA was amplified using Maximas SYBR Green/Fluorescein qPCR master mix (Thermo Fisher Scientific, USA).

The average threshold cycle (CT) values were normalized to the housekeeping gene (16s rRNA). The relative gene expression of the treated isolates was compared to that in the untreated ones according to the 2^−∆∆Ct^ method^27^. Primers are listed in Table S1^28,29^.

The antibiofilm activity of the fungal extract was further revealed using SEM. Briefly, cover slips in six-well plates were inoculated with *P. aeruginosa* bacteria suspension (10^7^ CFU/mL) and incubated overnight at 37 °C under two conditions: (i) medium alone (control, without the fungal extract) and (ii) medium supplemented with the fungal extract at 0.5 MIC values. After incubation, the coverslips were gently rinsed with sterile phosphate buffer to remove planktonic cells. For SEM preparation, samples were fixed in 2.5% glutaraldehyde, rinsed, and post-fixed in 1% osmium tetroxide. Dehydration was performed through a graded ethanol series, and the specimens were then dried, mounted on aluminium stubs, and sputter-coated with a gold-palladium layer. Imaging was performed using SEM (Hitachi, Japan), and representative micrographs were acquired^30,31^.

### Statistics

Quantitative data were designated as mean ± standard deviation (SD) using GraphPad Prism software (USA). The significance was referred to at *p* < 0.05. The Kruskal-Wallis test was employed for abnormally distributed quantitative variables to compare more than two studied groups, followed by Bonferroni for comparing each two groups. The Kaplan-Meier survival curve and the Chi-square for the log-rank test for significant survival were also used.

## Results

### *P. gladioli* fungus

The obtained fungal culture was identified by ITS region sequencing as *P. gladioli* (Fig. S1 and Table 1).

**Table 1:** Molecular identification of *P. gladioli* fungus by ITS region sequencing.

Identification	Highly similar Isolates	Identity %	Accession number
*Penicillium gladioli* isolate	*Penicillium gladioli* strain NRRL 939 18 S ribosomal RNA gene	98.89	PV893029

### GC-MS

**Fig. 2:**
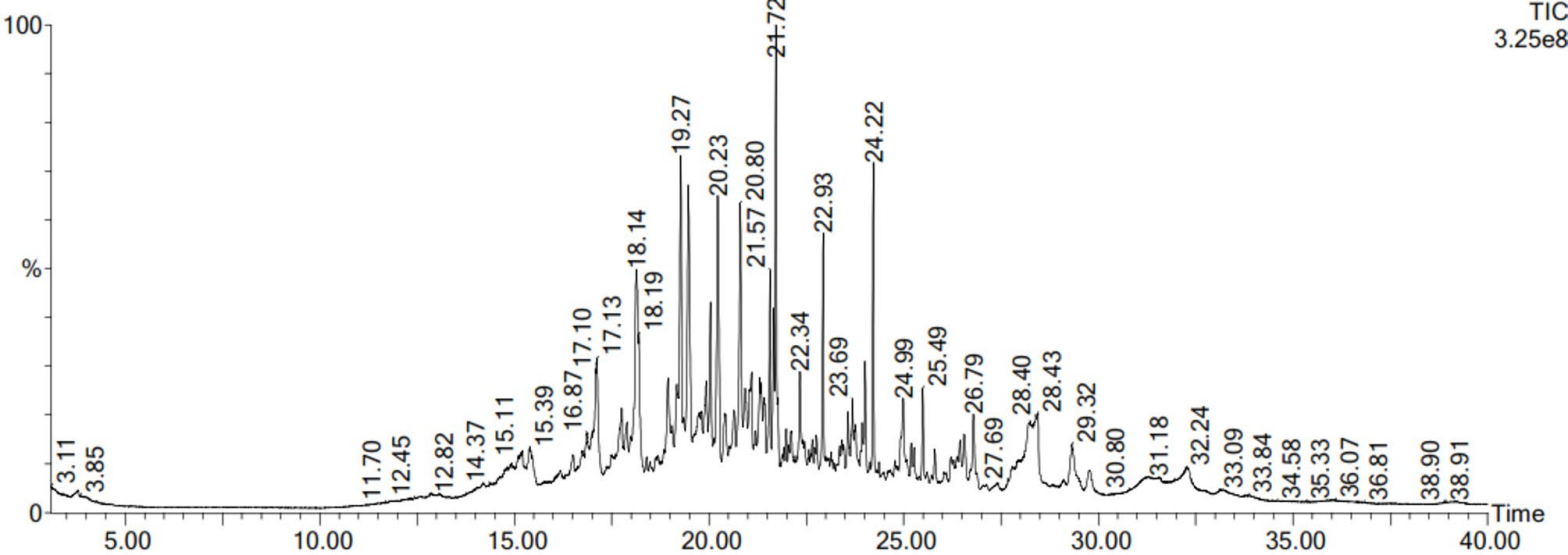
GC/MS spectrum of the *P. gladioli* fungal extract.

Bioactive metabolites were identified by GC-MS exploration, as GC-MS is considered one of the best procedures for differentiating the constituents of volatile matter, acids of alcohols, long chains, branched hydrocarbons, and esters. The compounds’ retention time (R_t_) and peak area percentage are presented in Table 2; Fig. 2.

**Table 2:** GC-MS investigation of the *P. gladioli* fungal extract.

No.	*R*_t_ (min)	Compound	Peak area %
1	14.8	1 1,2-Epoxyundecane	1.124
2	14.9	Nonanoic acid	0.884
3	15.1	Octanoic acid	1.9
4	15.3	Decanal	1.367
5	16.5	Cyclotetrasiloxane, octamethyl-	0.626
6	16.7	4-Octadecenal	0.915
7	16.8	Dodecalactone	1.026
8	17.1	2-Decenal, (Z)	3.568
9	17.7	Nonanoic acid	1.897
10	17.8	1,2-Epoxyundecane	1.052
11	18.1	Phenol, 2-methyl-5-(1-methylethyl)-	6.543
12	18.9	Naphthalene, 1,2,3,4,4a,7-hexahydro-1,6-dimethyl4-(1-methylethyl)	1.335
13	19.1	2-Norpinanol, 3,6,6-trimethyl-	0.806
14	19.2	2-Undecenal	3.155
15	19.4	Copaene	3.930
16	19.7	2-Dodecenal, (E)-	0.486
17	19.9	2-Undecanone, 6,10-dimethyl-	0.732
18	20	Dodecanal	1.504
19	20.2	Caryophyllene	3.891
20	20.4	Megastigma-4,6(E),8(Z)-triene	0.832
21	20.6	9-Tetradecenal, (Z)-	0.588
22	20.8	à-Caryophyllene	3.103
23	20.9	Z-10-Tetradecen-1-ol acetate	0.554
24	21	Naphthalene, 1,2,3,4,4a,5,6,8a-octahydro-7methyl-4-methylene-1-(1-methylethyl)-,(1à,4aà,8aà)-	1.144
25	21.3	8-Hexadecenal, 14-methyl-, (Z)-	0.672
26	21.34	à-Cubebene	0.656
27	21.4	Hexadecane, 1-chloro-	0.704
28	21.5	Tridecanal	1.344
29	21.6	Naphthalene, 1,2,4a,5,8,8a-hexahydro-4,7dimethyl-1-(1-methylethyl)-,[1 S-(1à,4aá,8aà)]	1.177
30	21.7	Naphthalene, 1,2,4a,5,8,8a-hexahydro-4,7dimethyl-1-(1-methylethyl)-,[1 S-(1à,4aá,8aà)]	3.708
31	22.1	2-Butyloxycarbonyloxy-1,1,10-trimethyl-6,9epidioxydecalin	0.415
32	22.3	Pentadecanoic acid	1.057
33	22.9	Tetradecanal	1.927
34	23.5	tau.-Cadinol	0.457
35	23.6	6,10,14-Trimethyl-pentadecan-2-ol	0.587
36	23.7	Naphthalene, 1,6-dimethyl-4-(1-methylethyl)-	0.495
37	23.9	1-Hexadecanol	0.549
38	24	2-Pentadecanone	1.054
39	24.2	Hexadecanal	2.588
40	24.9	Tetradecanoic acid	1.538
41	25.4	Tetradecanal	0.887
42	26.4	9-Tetradecenal, (Z)-	0.608
43	26.5	2-Heptadecanone	0.748
44	26.7	Octadecanal	1.081
45	28.4	n-Hexadecanoic acid	7.989
46	29.3	2(3 H)-Furanone, 5-dodecyldihydro-	1.678
47	29.7	2 H-Pyran-2-one, tetrahydro-6-nonyl-	0.694
48	31.2	Z-8-Methyl-9-tetradecenoic acid	3.313
49	32.2	Octadecanoic acid	4.138
50	33.1	cis-Vaccenic acid	1.046

### Parasitological results

#### Survival time and survival rate

**Fig. 3:**
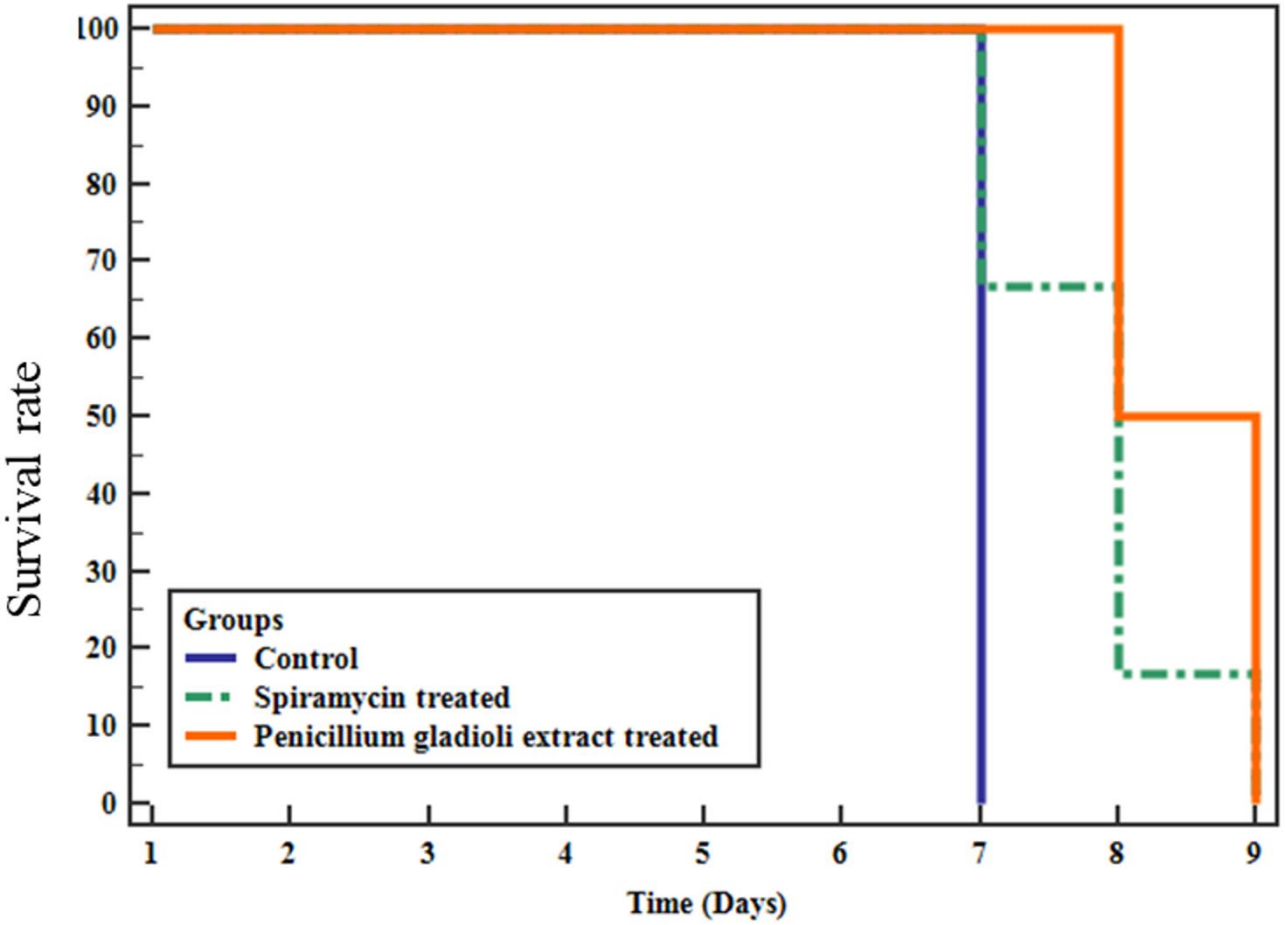
Kaplan-Meier survival curve of mice of the tested groups (*n* = 6 mice).

No mortality was detected on the day of scarification (the 6th day post-infection) in all groups. All six mice in the control group (group I) died on the 7th day. In the spiramycin-treated group (group II), four of the six mice died on the 7th day (66.7% survived mice) and one on the 8th day (16.7% survived mice) with a mean of 7.83 days. In the *P. gladioli* extract-treated group (group III), none of the six mice died on the 7th day (100% survived mice), and three on the 8th day (50% survived mice) with a mean of 8.5 days. The prolonged survival time in group III was substantial (*p* < 0.001) in comparison to the non-treated control group (group I) and the spiramycin-treated group (group II) (Fig. 3, Table S2).

#### Mean number of tachyzoites of *T. gondii*.

In comparison to group I (mean ± SD = 545.5 ± 343.0), treated groups displayed a noteworthy decline (*p* < 0.001) in the tachyzoites count, with a mean ± SD of 22.2 ± 21.4 and 36.3 ± 26.4 with a reduction of 95.9% and 93.3% in group II (spiramycin) and group III (*P. gladioli* extract), respectively (Table 3).

**Table 3:** Number of the tachyzoites (×10^4^)/ml in the peritoneal fluid (*n* = 6).

Tachyzoites number (×10^4^)	Group I	Group II	Group III	H	*p*-value
Min.–Max.	64.0-980.0	5.0–64.0	17.0–86.0		
Mean ± SD	545.5 ± 343.0	22.2 ± 21.4	36.3 ± 26.4	11.477*	0.003*
SEM	140.0	8.7	10.8		
Median	548.0	17.5	26.0		
Tachyzoites reduction rate		95.9	93.3		
Pairwise comparison	*p*_1_ = 0.001*, *p*_2_= 0.021*, *p*_3_ =0.316				

*Group I is the control infected non-treated group, group II is the infected, spiramycin-treated group and group III is the infected *P. gladioli* extract-treated group. Min.: minimum, Max.: maximum, SD: standard deviation, SEM: standard error of the mean. H: Kruskal–Wallis test. *p*_1_: *p-*value for comparing between group I and group II, *p*_2_: *p*-value for comparing between group I and group III, *p*_3_: *p*-value for comparing between group II and group III. The significance was established at *p* ≤ 0.05.

### SEM

**Fig. 4:**
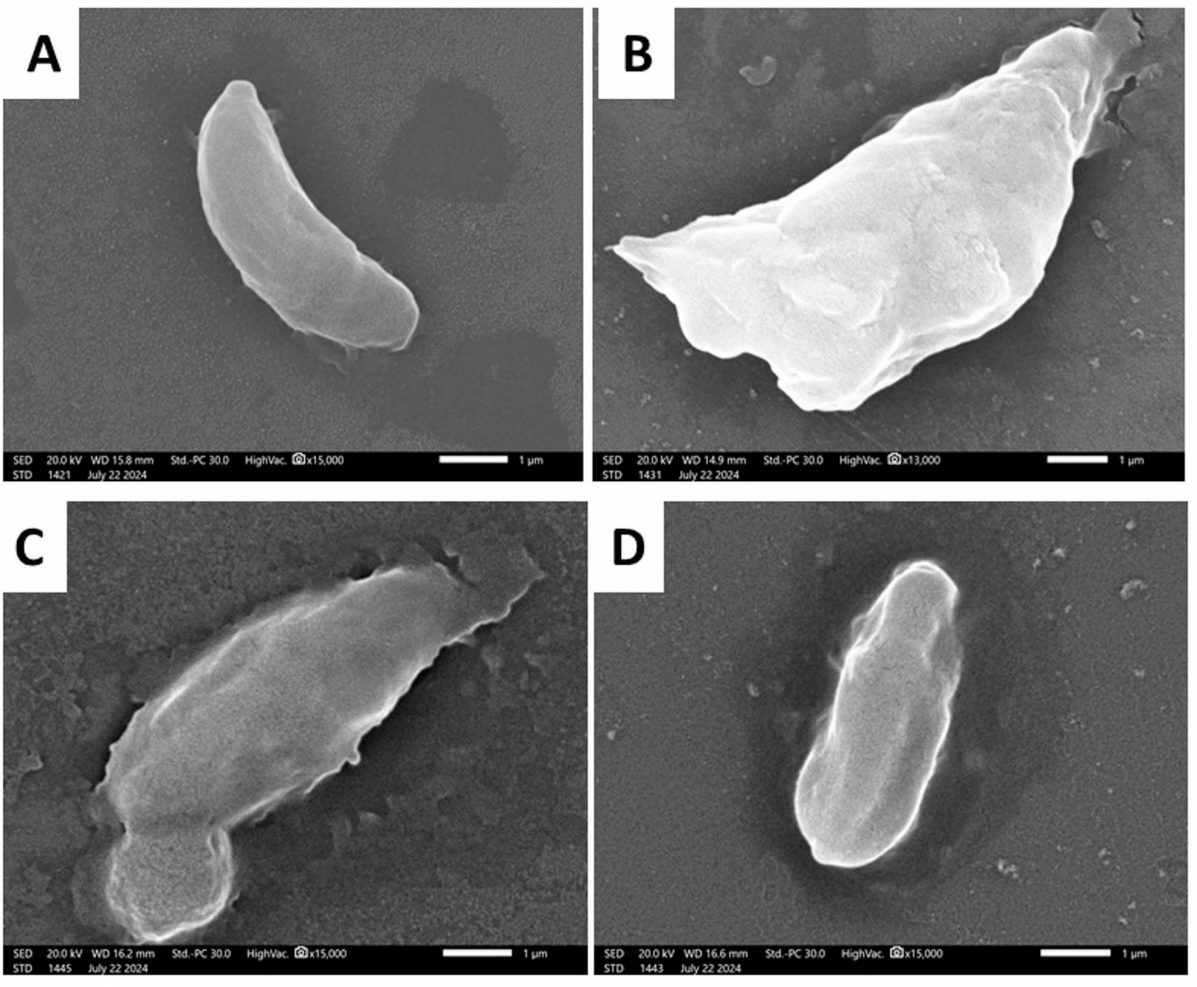
The scanning electron micrograph of *T. gondii* tachyzoites (×15000). Tachyzoites in group I (Positive control) showed a normal size, shape, and surface (**A**). Group II (Spiramycin group) showed an increase in the size with coarse surface ridges (**B**). Group III (Fungal extract group) showed an increased size with surface irregularities, such as a tear in the upper end and a bulge in the lower end (**C**). In addition, group III (**D**) revealed surface ridges and depressions.

SEM of *T. gondii* tachyzoites attained from the peritoneal fluid of the untreated group (group I) exposed normal-sized crescent-like tachyzoites with smooth surfaces (Fig. 4A). In contrast, group II, treated with spiramycin, showed an increase in the tachyzoites’ size in the form of ballooning with surface irregularities and coarse ridges (Fig. 4B). Likewise, in the *P. gladioli* extract-treated group (group III), tachyzoites increased in size with surface irregularities, a tear in the upper end, and a bulge in the lower end (Fig. 4C). Tachyzoites in Fig. 4D showed surface ridges and depressions.

### Histopathological and immunohistochemical results

**Fig. 5:**
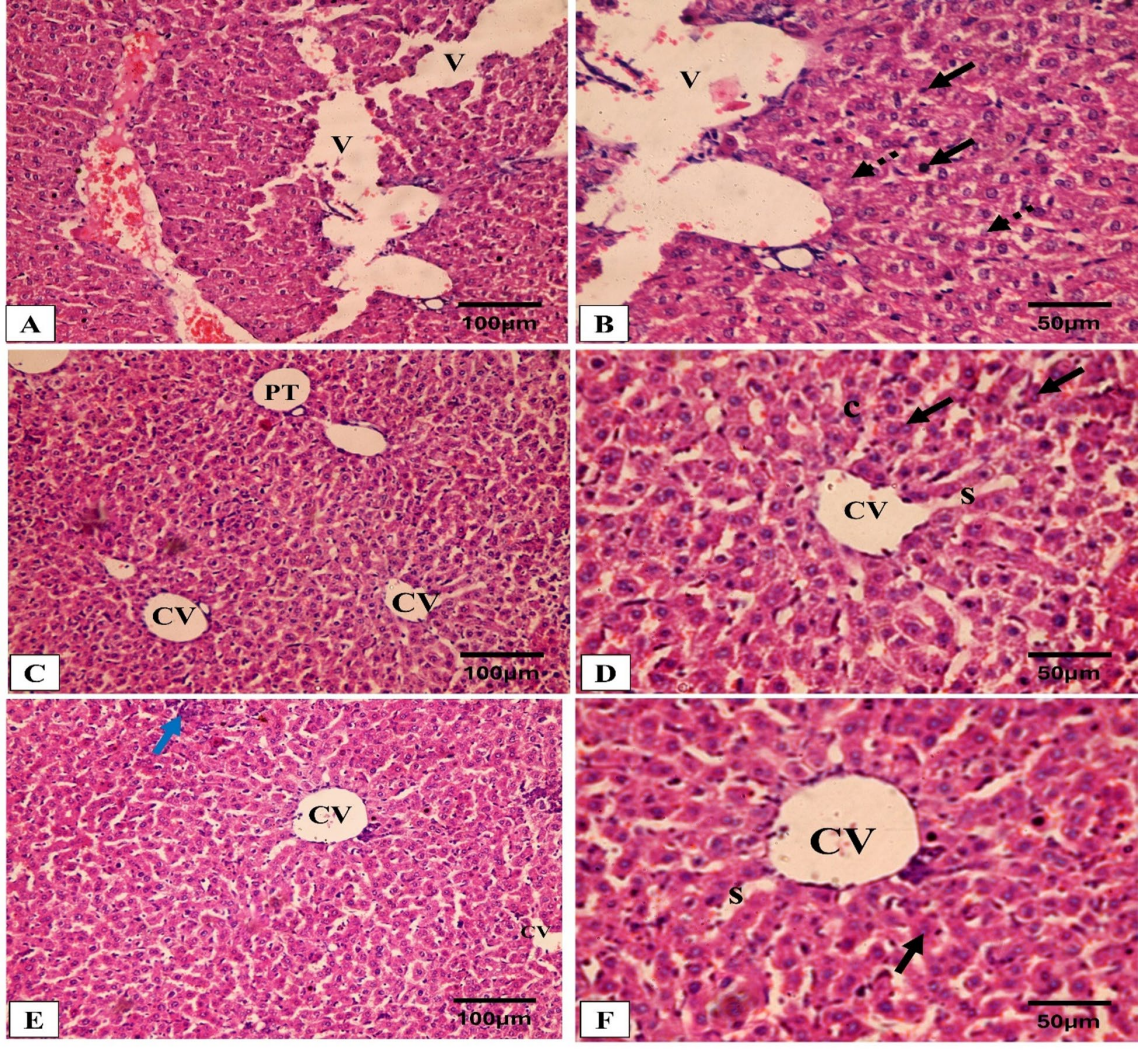
Analysis of liver architecture by H&E staining. (**A**,**B**) Group I (Positive control) shows a loss of the normal hepatic architecture, compressed hepatic sinusoids, multiple pyknotic nuclei (black arrow), multiple karyolitic nuclei (dashed black arrow), and diffuse vacuolar degeneration of hepatocytes with multiple large vacuoles (V) and ballooned hepatocytes. (**C**–**F**) Groups II (Spiramycin group) and III (Fungal extract group) show marked improvement and regaining of the normal hepatic structure in the form of organized hepatic architecture with hepatic cords (c) radiating from the central vein (CV) and separated by the hepatic sinusoids (S) with pericentral zone and midzone hepatocytes. Polyhedral hepatocytes appear with rounded vesicular nuclei and granular eosinophilic cytoplasm (black arrow) separated by sinusoids (S) which are lined with endothelial cells and Kupffer cells. Also, portal triad (PT) and some inflammatory cells (blue arrow) can be seen in group III (Fungal extract group) (**E**).

**Fig. 6:**
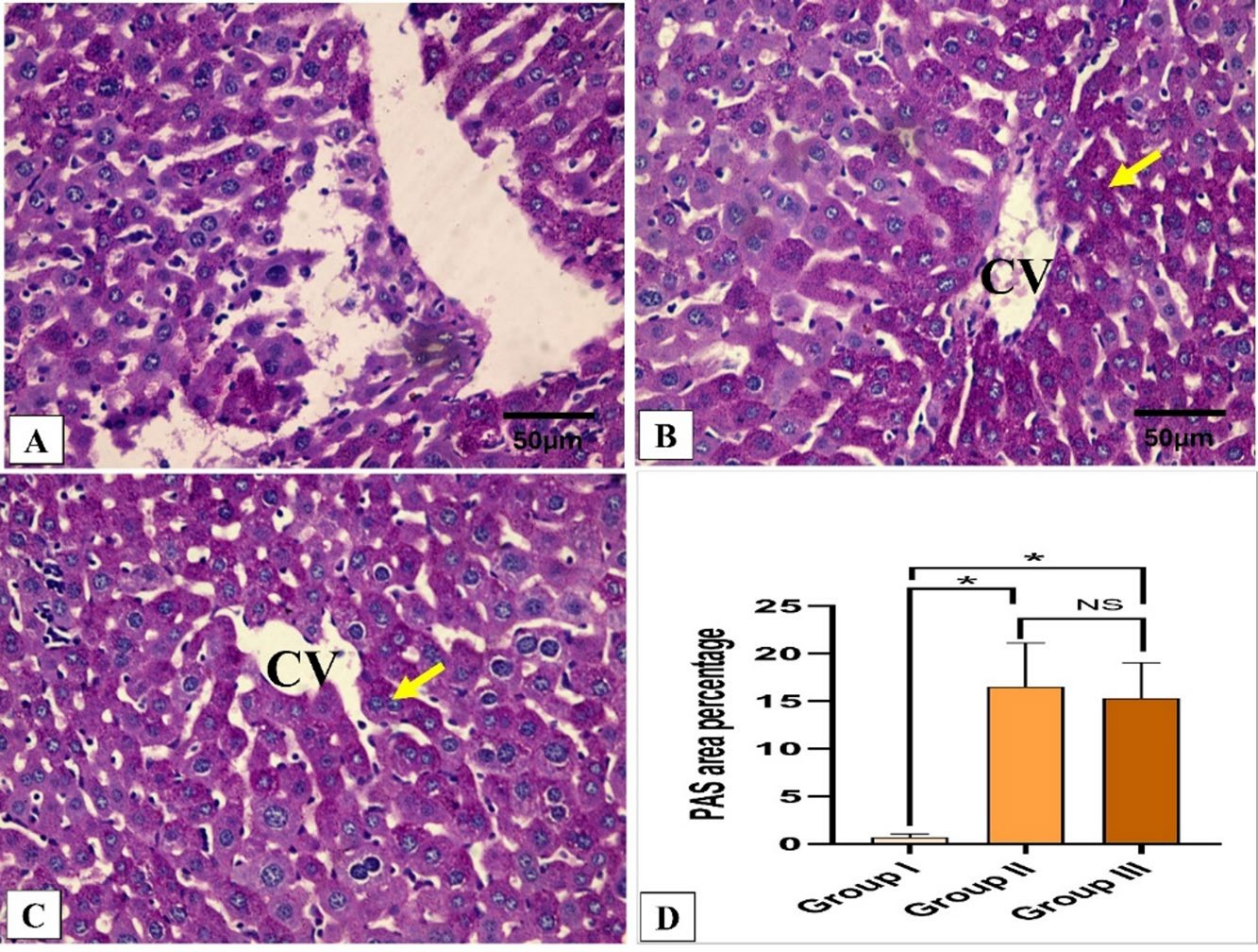
Analysis of liver glycogen by PAS staining. (**A**) Group I (Positive control) expresses weak PAS-positive cells. (**B**,**C**) Groups II (Spiramycin group) and III (Fungal extract group) express a strong PAS cytoplasmic stain (glycogen) in the hepatocytes around the central veins. (**D**) Area percentage of PAS-positive cells in all groups. The single asterisk designates a substantial (*p* < 0.05) difference, and the abbreviation (NS) designates a non-significant (*p* > 0.05) difference where *n* = 10. (PAS × 400, scale bar = 50 μm).

**Fig. 7:**
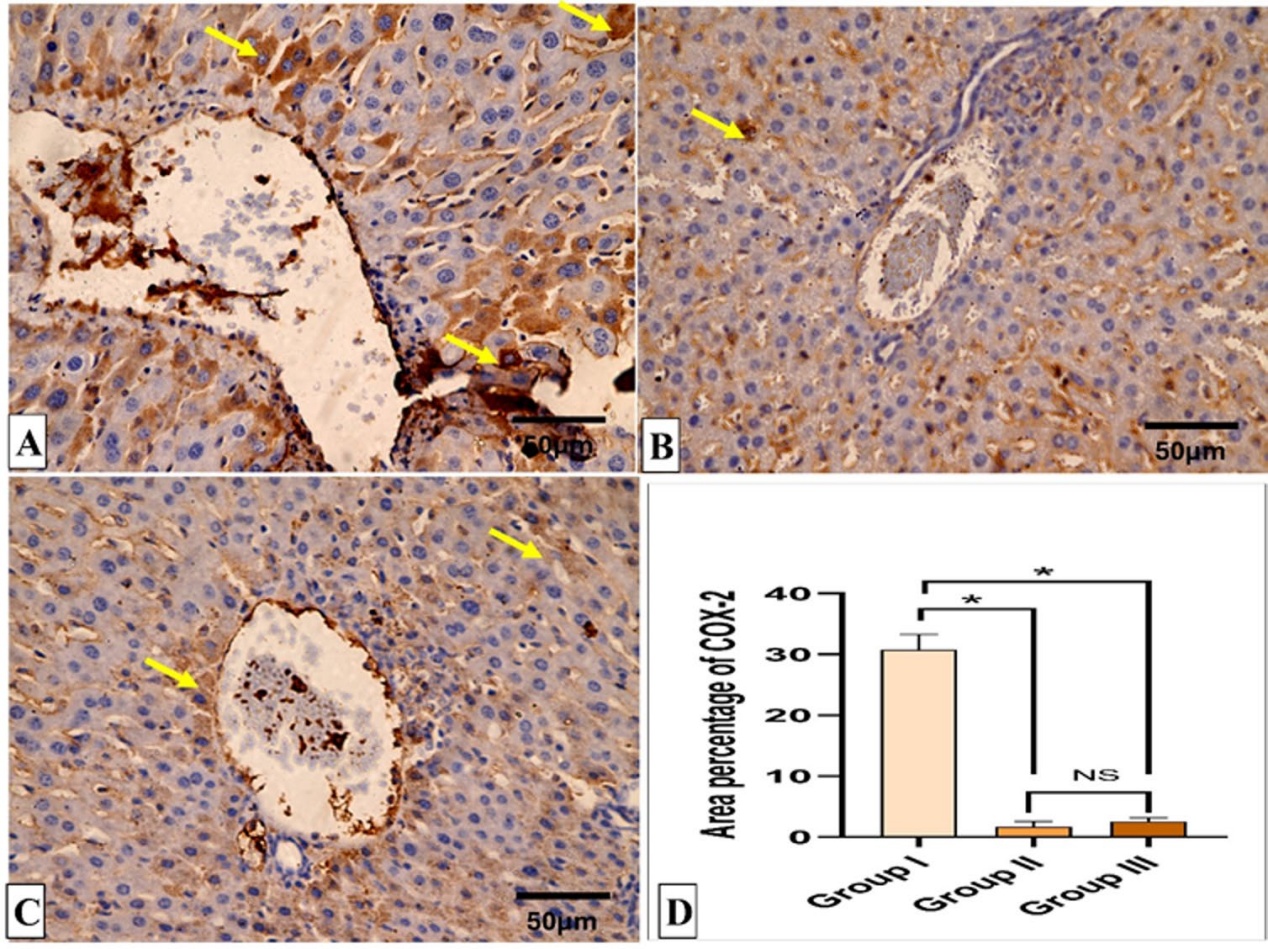
COX-2 immunohistochemical staining analysis of liver sections. (**A**) Group I (Positive control) expresses a strong positive COX-2 reaction in the form of brown stain within the cytoplasm of hepatocytes (yellow arrows) with a significant difference from both groups II (Spiramycin group) and III (Fungal extract group). (**B**,**C**) Groups II (Spiramycin group) and III (Fungal extract group) show few COX-2 positive cells (yellow arrow) and a negative reaction in most cells with non-significant difference with each other. (**D**) Area percentage of COX-2 immune reaction in all groups. The single asterisk designates a substantial (*p* < 0.05) difference, and the abbreviation (NS) designates a non-significant (*p* > 0.05) difference where *n* = 10. (COX-2 × 400, scale bar = 50 μm).

**Fig. 8:**
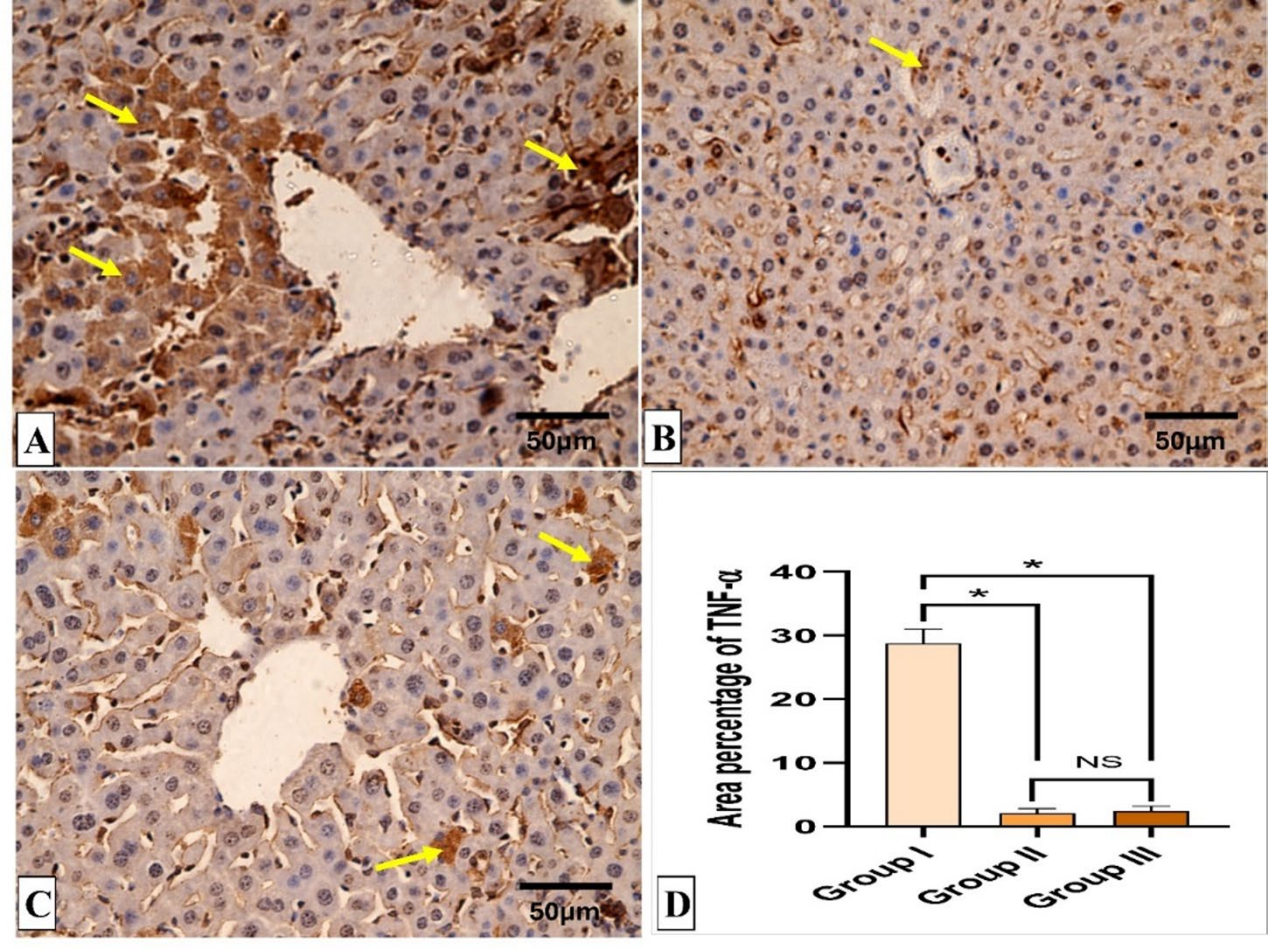
TNF-α immunohistochemical staining analysis of liver sections. (**A**) Group I (Positive control) expresses a strong positive TNF-α reaction in the form of brown stain within the cytoplasm of hepatocytes (yellow arrows) with a significant difference from both group II (Spiramycin group) and III (Fungal extract group). (**B**,**C**) Groups II (Spiramycin group) and III (Fungal extract group) show few TNF-α positive cells (yellow arrow) and negative reaction in the most of cells with a non-significant difference from each other. (**D**) Area percentage of TNF-α immune reaction in all groups. The single asterisk designates a substantial (*p* < 0.05) difference, and the abbreviation (NS) designates a non-significant (*p* > 0.05) difference where *n* = 10. (TNF-α × 400, scale bar = 50 μm).

**Fig. 9:**
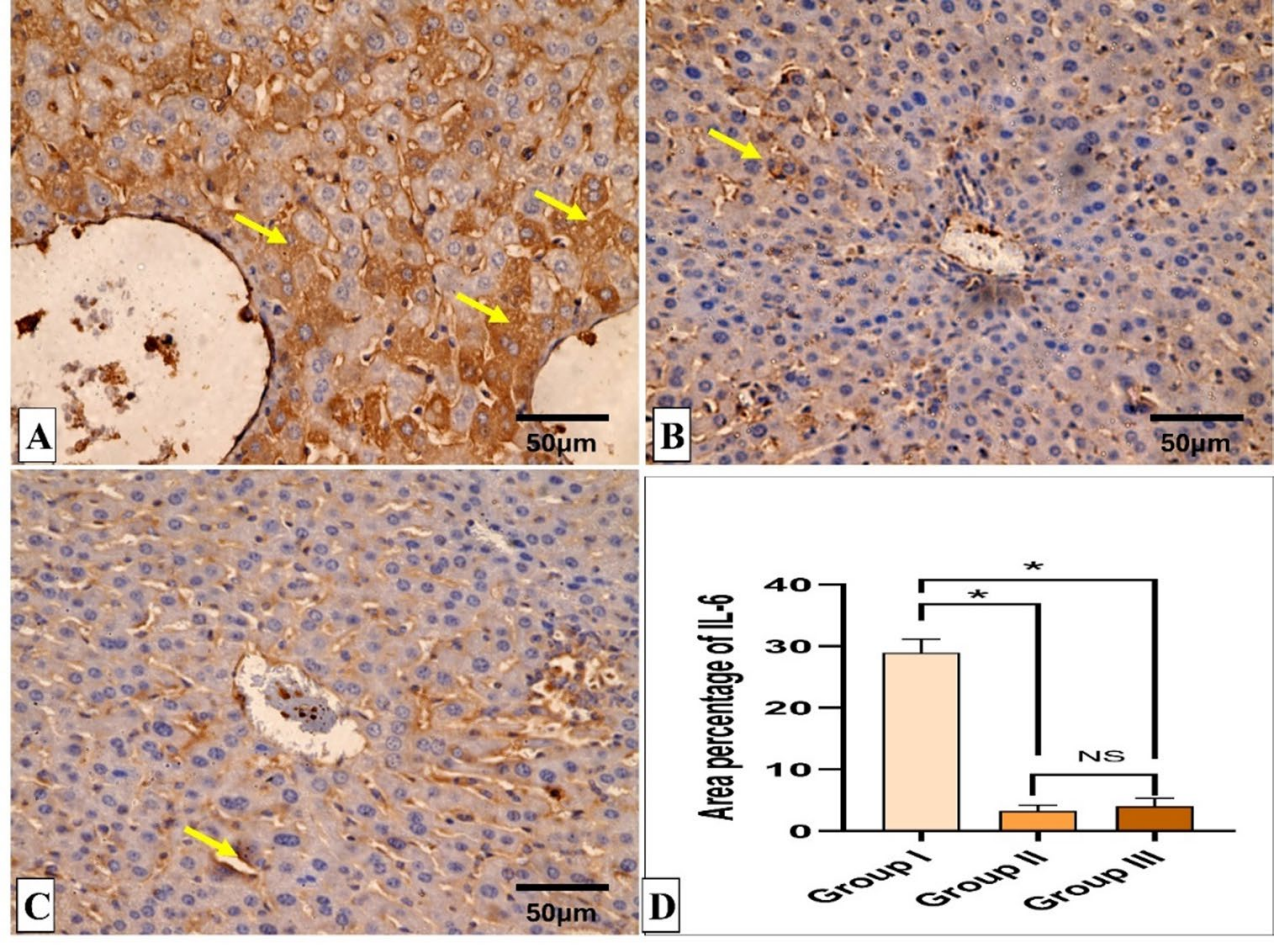
IL-6 immunohistochemical staining analysis of liver section. (**A**) Group I (Positive control) expresses a strong IL-6 positive reaction in the form of brown stain within the cytoplasm of hepatocytes (yellow arrows) with a significant difference from both groups II (Spiramycin group) and III (Fungal extract group). (**B**,**C**) Groups II (Spiramycin group) and III (Fungal extract group) show few IL-6 positive cells (yellow arrow) and a negative reaction in most cells with a non-significant difference from each other. (**D**) Area percentage of IL-6 immune reaction in all groups. The single asterisk designates a substantial (*p* < 0.05) difference, and the abbreviation (NS) designates a non-significant (*p* > 0.05) difference where *n* = 10. **(**IL-6 × 400, scale bar = 50 μm).

**Fig. 10:**
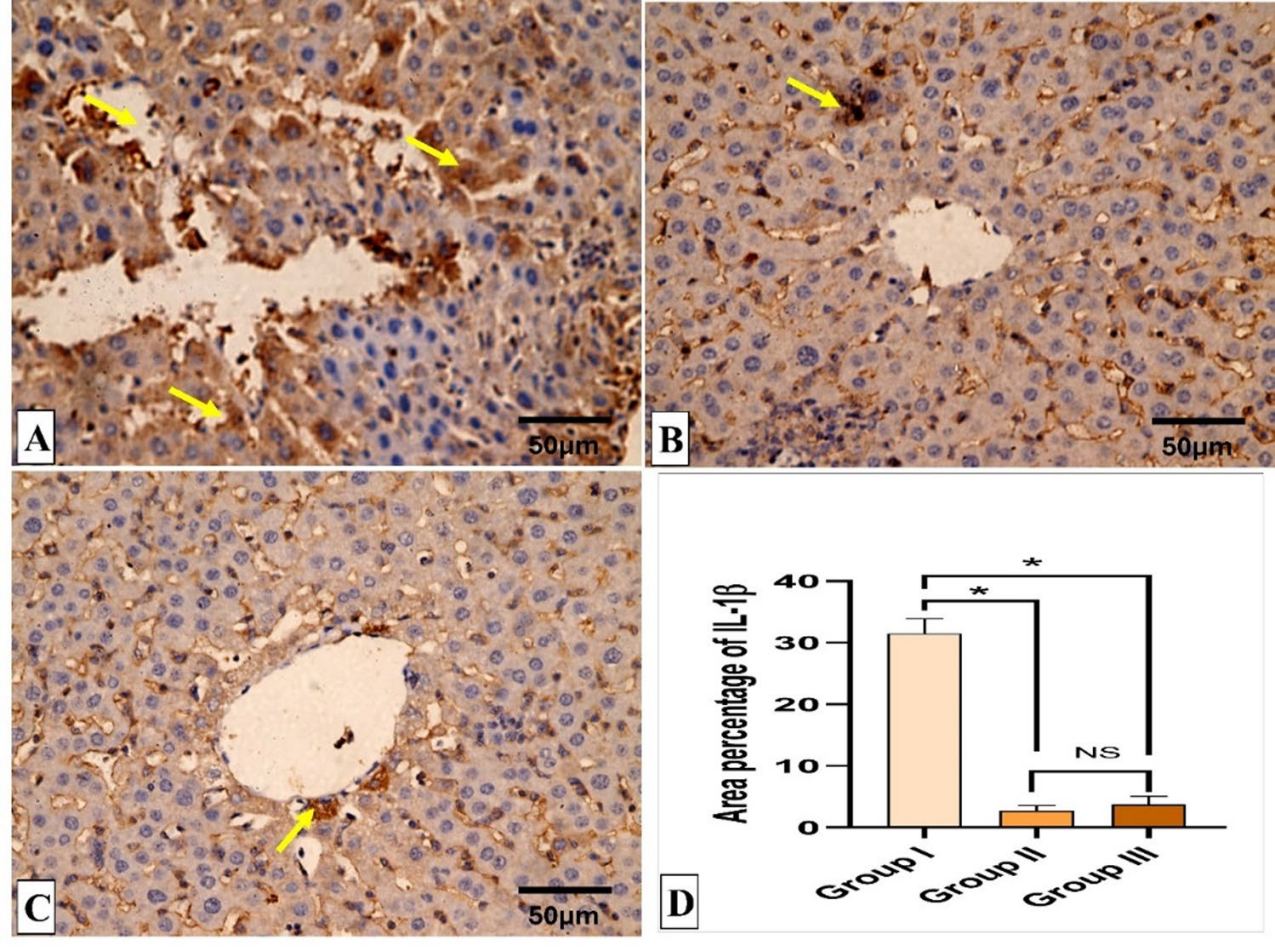
IL-1β immunohistochemical staining analysis of liver section. (**A**) Group I (Positive control) expresses a strong IL-1β positive reaction in the form of brown stain within the cytoplasm of hepatocytes (yellow arrows) with a significant difference from groups II (Spiramycin group) and III (Fungal extract group). (**B**,**C**) Groups II (Spiramycin group) and III (Fungal extract group) show few IL-1β positive cells (yellow arrow) and a negative reaction in the most of cells with a non-significant difference from each other. (**D**) Area percentage of IL-1β immune reaction in all groups. The single asterisk designates a substantial (*p* < 0.05) difference, and the abbreviation (NS) designates a non-significant (*p* > 0.05) difference where *n* = 10. (IL-1β × 400, scale bar = 50 μm).

Figures 5 and 6 reveal the liver histological features of the different groups. Figures 7, 8, 9 and 10 also reveal COX-2, TNF-α, IL-6, and IL-1β immunohistochemical reactions.

### Colorimetric results

**Fig. 11:**
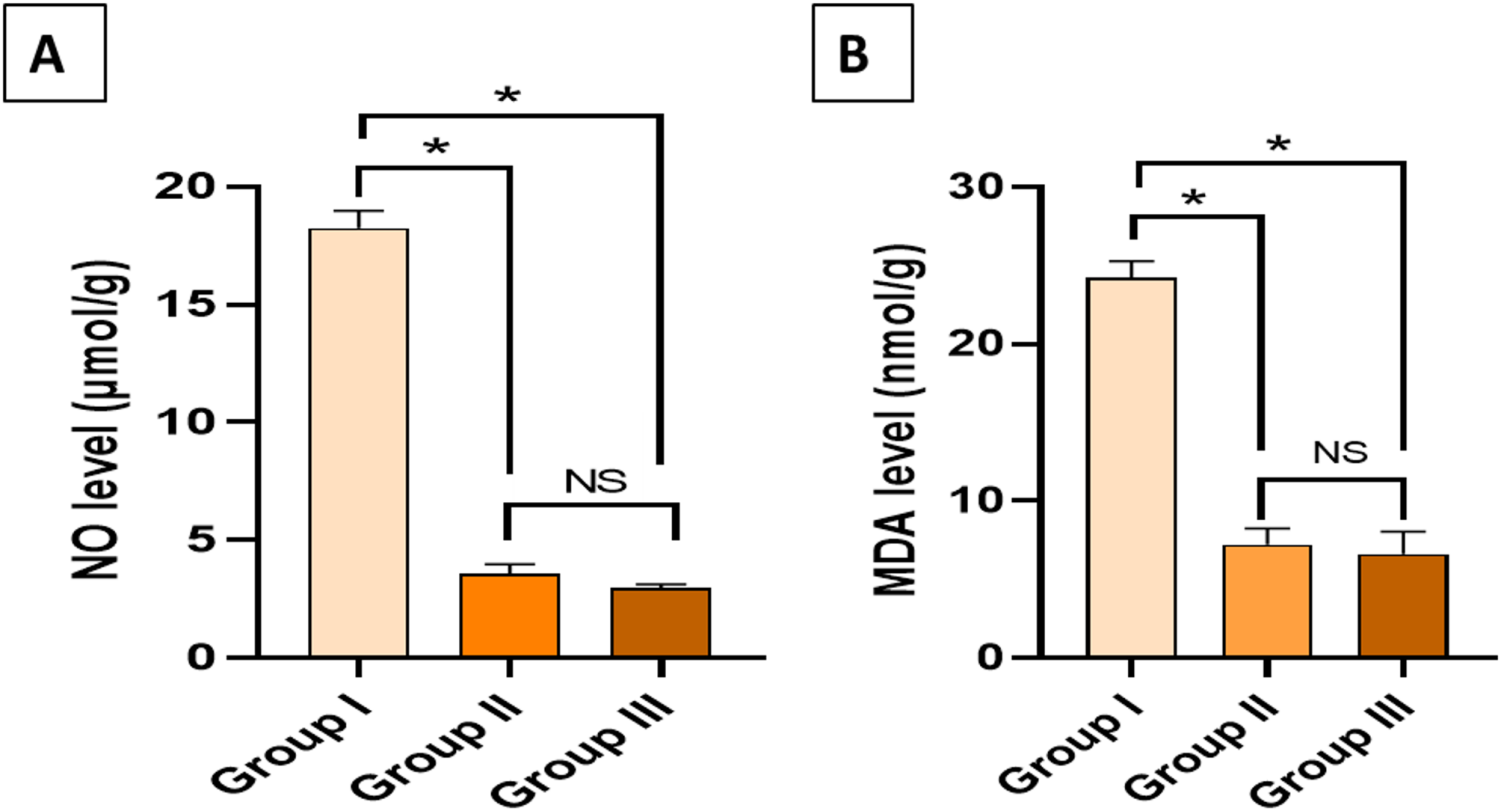
(**A**) NO level and (**B**) MDA level in the liver. The sign (*) denotes a noteworthy difference, and (NS) denotes a non-significant difference at *p* > 0.05.

As oxidative stress markers, NO and MDA levels were noticeably diminished (*p* < 0.05) in group III compared to group I, as exposed in Fig. 11. This outcome designates the antioxidant consequence of the fungal extract, which helps it in the anti-toxoplasma consequence to alleviate the detrimental effects of the infection.

### Antibacterial potential of the fungal extract on *P. aeruginosa* isolates

**Fig. 12:**
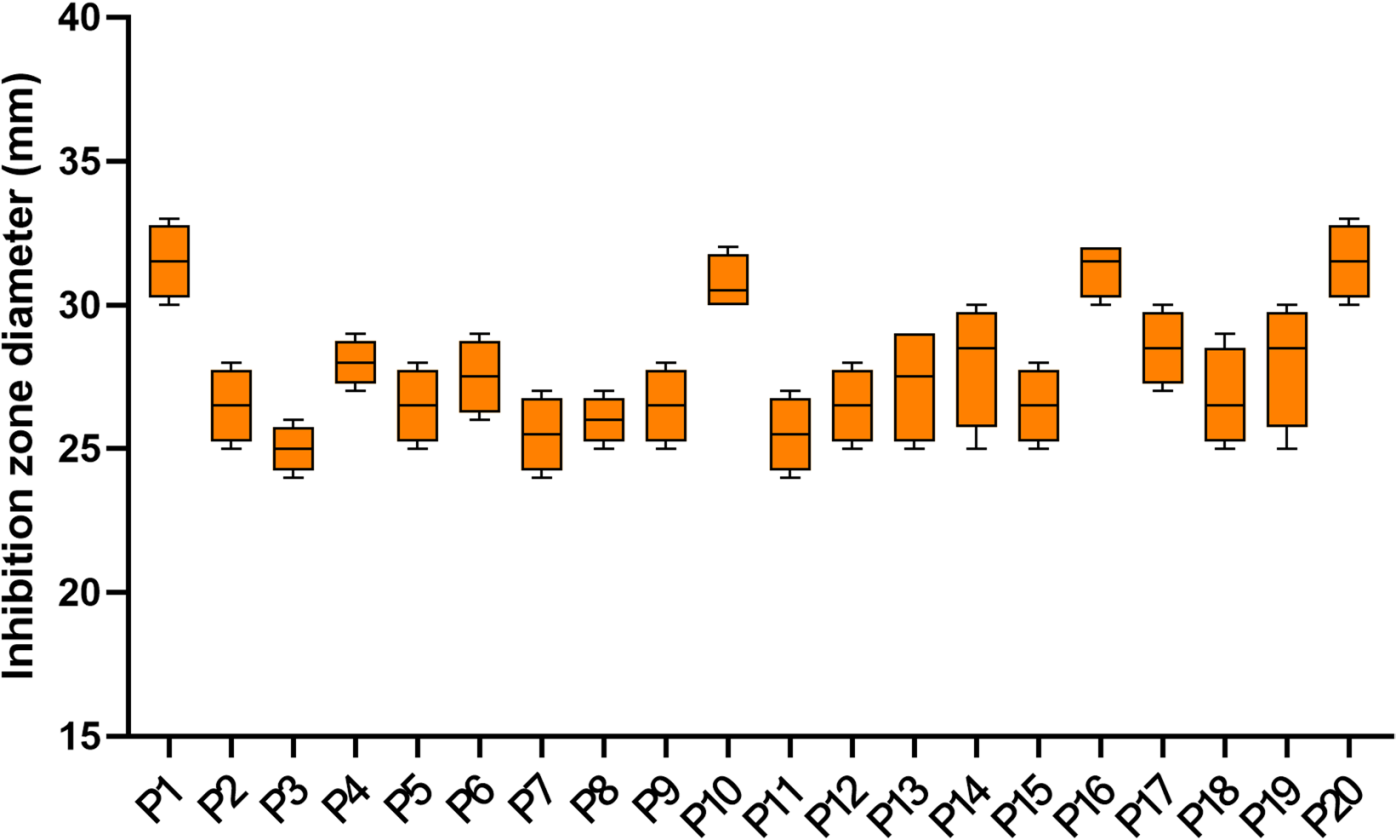
Inhibition zones of the fungal extract on *P. aeruginosa* isolates (*n* = 3).

The fungal extract exposed inhibition zones around the wells, as shown in Fig. 12. The MICs of the fungal extract ranged from 64 to 512 µg/ml (Table 4).

**Table 4:** Minimum inhibitory concentrations (MICs) of the fungal extract on *P. aeruginosa* isolates.

Isolate code	MIC (µg/ml)	Isolate code	MIC (µg/ml)
P1	64	P11	64
P2	128	P12	128
P3	512	P13	512
P4	256	P14	128
P5	64	P15	64
P6	128	P16	64
P7	256	P17	512
P8	512	P18	128
P9	512	P19	256
P10	64	P20	128

### Antibiofilm action

About 65% of *P. aeruginosa* isolates were able to form biofilm strongly or moderately. Remarkably, the fungal extract considerably declined this percentage to 20% (Table 5).

**Table 5:** The consequence of *P. gladioli* extract on the biofilm formation by *P. aeruginosa* isolates.

Isolate code	Without fungal extract	With fungal extract	Isolate code	Without fungal extract	With fungal extract
P1	Moderate	Weak	P11	Non-forming	Non-forming
P2	Moderate	Non-forming	P12	Moderate	Non-forming
P3	Strong	Non-forming	P13	Strong	Moderate
P4	Weak	Non-forming	P14	Strong	Moderate
P5	Moderate	Weak	P15	Weak	Weak
P6	Moderate	Non-forming	P16	Moderate	Non-forming
P7	Strong	Moderate	P17	Weak	Non-forming
P8	Strong	Weak	P18	Non-forming	Non-forming
P9	Non-forming	Non-forming	P19	Strong	Moderate
P10	Non-forming	Non-forming	P20	Moderate	Weak

**Fig. 13:**
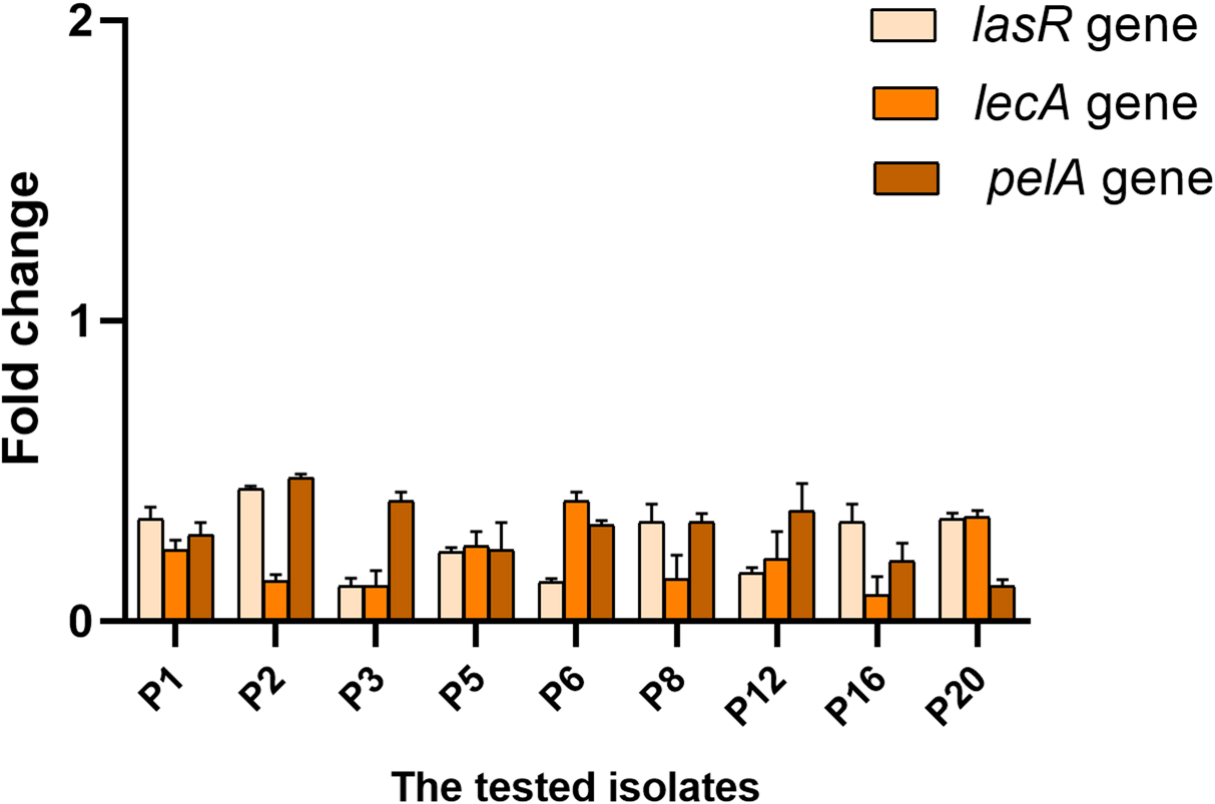
Fold change of the biofilm formation genes in the tested isolates with the *P. gladioli* extract (*n* = 3).

Figure 13 exposes the result of *P. gladioli* on the biofilm gene expression, as it downregulated the biofilm-related genes in 45% of the isolates.

**Fig. 14:**
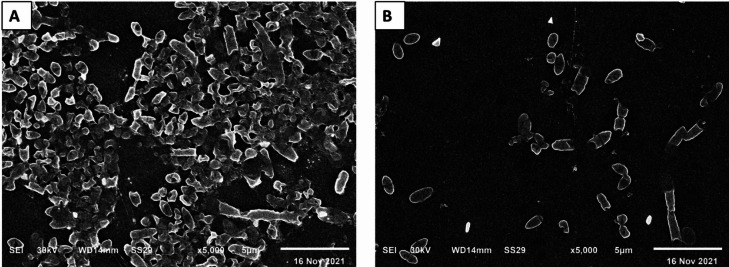
Scanning electron micrograph of a representative *P. aeruginosa* isolate (**A**) without and (**B**) with the fungal extract.

SEM was utilized to illuminate the consequence of the fungal extract on the bacterial biofilm. As shown in Fig. 14, a noteworthy decline in the number of the biofilm-forming isolates was found after treatment with *P. gladioli* extract.

## Discussion

Soil is regarded as one of the most appropriate environments for the growth of many microbes, like bacteria and fungi. Here, the saprophytic fungus, *P. gladioli*, was isolated and identified from a soil sample from Tanta, Egypt. We investigated its anti-toxoplasma and antibacterial action as the fungal extract represents a sustainable and eco-friendly source of various active compounds.

A remarkable number of the natural product-derived drugs originate from microbes or plants^32^. Natural products from microbes, like fungi, are a significant source of many antibacterial and anti-parasitic agents. This is attributed to the abundant diversity of the fungal species and their numerous secondary metabolites. They are better than plants as they don’t necessitate different land and water resources and can yield a vast biomass with a low cost^33^.

GC-MS is an efficient method for recognizing constituents in natural extracts^34^. Thus, we employed GC-MS to elucidate the bioactive chemicals in the ethyl acetate fungal extract, revealing the presence of 50 compounds. Among the detected peaks, n-hexadecanoic acid showed the largest relative peak area (7.989% of the total ion chromatogram), followed by phenol, 2-methyl-5-(1-methylethyl) (6.543%). Antibacterial and anti-toxoplasma activities of the fungal extract may be related to these active compounds. Kalariya et al.^35^ documented the antibacterial, antifungal, and anti-parasitic actions of naphthalene derivatives produced by *P. gladioli*. Similarly, Oliveira et al.^36^. stated the anti-leishmanial activity of naphthalene derivatives. Donger et al.^37^ found that undecenal possessed antimicrobial, antioxidant, and antidiabetic activities. Shaaban et al.^38^ have also designated an antimicrobial consequence of hexadenoic acid against multidrug-resistant bacteria. Also, it was demonstrated by Lalthanpuii et al.^39^ that hexadecanoic acid was effective against the tapeworm *Raillietina echinobothrida.*

Based on the inadequate therapeutic choices, many adverse effects, and the risk of emergence of many resistant parasites, several researchers have considered medicinal natural extracts as potential safe new drugs for treating toxoplasmosis^40,41^. Various bioactive compounds from the fungi extract have demonstrated diverse pharmacological effects against different microbes^42^. Here, the anti-parasitic effect of *P. gladioli* on *T. gondii* was elucidated in mice. The longest survival time was reported in the *P. gladioli* extract-treated group (group III) with a mean of 8.5 days, which was statistically noteworthy (*p* < 0.05) in comparison to the non-treated control (group I) and spiramycin-treated group (group II). Almallah et al.^18^. documented a boosted survival rate of mice infected with *T. gondii* treated with probiotics as a safe, natural tested agent.

Regarding the parasite load, the treated groups exhibited a noteworthy decline (*p* < 0.05) in the mean count of tachyzoites in the peritoneal fluid compared to group I. The significant decline was 93.3% in the *P. gladioli* extract-treated group (group III) with a mean ± SD of 36.3 ± 264 in comparison to the group I with a mean ± SD of 545.5 ± 343.0. This reduction in the percentage of parasite count is consistent with the results of different recent investigations accomplished on *T. gondii* tachyzoites^4,17–19,43–45^.

Several bioactive agents formed by saprophytic fungi can lessen reactive oxygen species levels, subsequently decreasing inflammation^46–49^. The main cause of the mice’s bad clinical condition and death is the severe inflammation produced in the vital organs by the massive parasite replication^46^. Here, the fungal extract-treated group showed a noteworthy diminishing in the immunostaining of COX-2, TNF-α, IL-6, and IL-1β, which are inflammatory markers. This lessening in the inflammatory mediators could be related to the decrease in the parasite-induced tissue damage. The fungal extract could have anti-inflammatory potential on its own, but this point necessitates further studies to establish whether the extract exerts a direct immunomodulatory effect. Previous reports have described the potential anti-inflammatory action of fungal extracts^50,51^.

SEM elucidation of the tachyzoites in the peritoneal fluid of *P. gladioli* extract-treated group (group III) showed a distortion in the tachyzoites’ shape and irregularities of their surface in the form of bulges, ridges, and depressions. These changes demonstrated the effect of the oral inoculation of the fungal extract on the tachyzoites in the peritoneal fluid. The adequate bioavailability of the extract allowed its presence in tissue in sufficient amounts that could affect the parasites^15^. Additionally, the surface distortion at the ultrastructural level could have reduced the reproduction of the tachyzoites, which was a principal cause of the significant decrease in the parasite load^52^.

The *P. gladioli* fungal extract revealed antibacterial action on the tested *P. aeruginosa* isolates. This could be attributed to the bioactive chemicals in the fungal extract. Ganesan et al.^53^ described the antibacterial action of n-hexadecanoic acid on *Staphylococcus aureus*, *Bacillus subtilis*, *Escherichia coli*, and *Klebsiella pneumoniae*. Also, Sajayan et al.^54^. documented the antibacterial and antibiofilm action of n-hexadecanoic acid from marine sponge-associated bacteria *Bacillus subtilis*. Many studies have documented the antibacterial action of phenolic compounds like phenol, 2-methyl-5-(1-methylethyl)^55–57^. The chief mechanism of action of phenolic compounds is the disruption of the bacterial membranes.

**Fig. 15:**
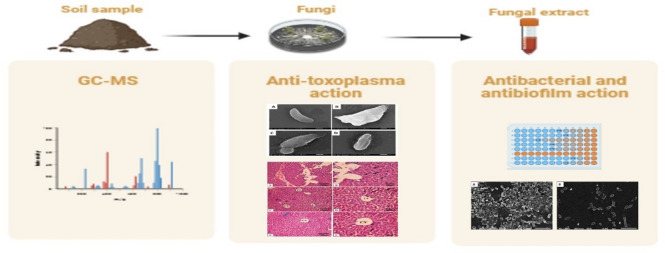
Representation of the experiments performed in this study.

Biofilms are reported to be involved in more than 60% of human infections^58^. Therefore, substantial research has focused on finding antibiofilm compounds. In *P. aeruginosa*, biofilm formation relies mostly on the communication between cells by quorum-sensing (QS) controlled by QS genes like *las*R^59^. Also, *P. aeruginosa* can produce a cytotoxic lectin expressed by the lecA gene in the biofilm-forming cells. In addition, the *lecA* gene is important in the biofilm architecture as it is involved in the extracellular matrix, which controls the biofilm structural integrity. The *pelA* gene plays a noteworthy role in forming the carbohydrate-rich structure in biofilm^60^. Here, we inspected the antibiofilm outcome of the fungal extract by crystal violet, SEM, and qRT-PCR. It elucidated a remarkable antibiofilm action as it downregulated the biofilm-related genes in 45% of the isolates. One of the limitations in our study is employing GC-MS analysis for chemical profiling of the fungal extract, as it is limited to volatile and semi-volatile constituents, and thus other non-volatile compounds with potential biological activity may not have been detected. The individual compounds in the crude fungal extract will be elucidated for their own anti-toxoplasma and antibacterial potentials in our future studies. Figure 15 represents the experiments conducted in this study.

## Conclusion

The current work suggests that *P. gladioli* could serve as a new alternate treatment for acute toxoplasmosis and *P. aeruginosa* infections. It revealed a detrimental impact on the parasite in vivo and bacteria in vitro. It could also be investigated on other parasites and bacterial species. Future clinical studies should also be performed on this saprophytic fungus to illuminate its effectiveness in clinical settings.

## Supplementary Information

Below is the link to the electronic supplementary material.

Supplementary Material 1

## Data Availability

Data is provided within the manuscript and the supplementary information.
