# Supplementary material for: Multifunctional bioactivity of eco-friendly Penicillium gladioli extract against Toxoplasma gondii and Pseudomonas aeruginosa
Source: Sci Rep. 2025 Nov 26;15:42297. doi: 10.1038/s41598-025-23921-z (PMC12660671; doi:10.1038/s41598-025-23921-z)
Supplement: Supplementary file 1 — Supplementary Material 1 [file 41598_2025_23921_MOESM1_ESM.docx]

**Table S1.** Sequences of the utilized primers

| **Gene** | **Forward Primer (5′ to 3′)** | **Reverse Primer (5′ to 3′)** |
| --- | --- | --- |
| *las*R | AAGTGGAAAATTGGAGTGGAG | GTAGTTGCCGACGATGAAG |
| *lec*A | CACCATTGTGTTTCCTGGCGTTCA | AGAAGGCAACGTCGACTCGTTGAT |
| *pel*A | AAGAACGGATGGCTGAAGG | TTCCTCACCTCGGTCTCG |
| 16S rRNA (housekeeping gene) | ACGCAACTGACGAGTGTGAC | GATCGCGACACCGAACTAAT |


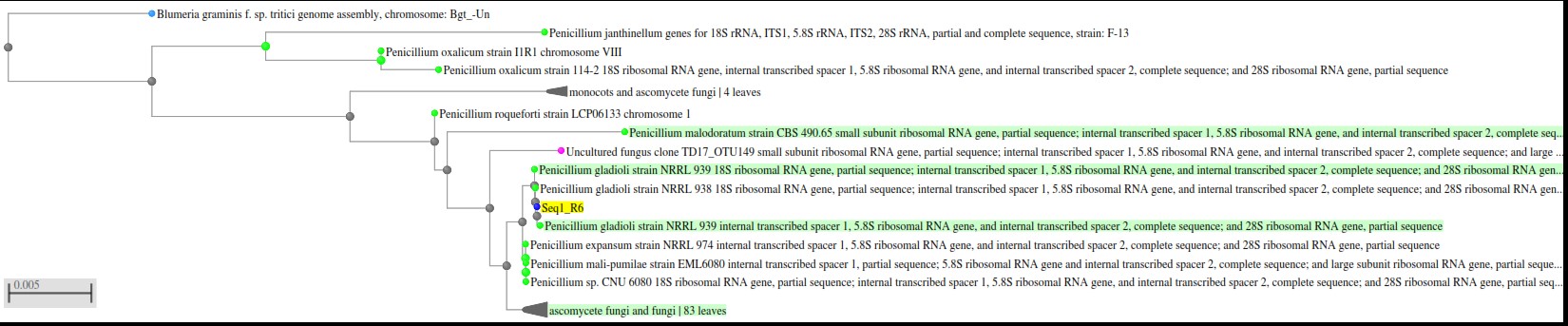


**Figure S1.** Phylogenetic tree of *P.* *gladioli* fungus (with yellow highlight) based on ITS region sequencing.

**Table S2.** Survival time and survival rate of mice.

| **Groups** | **Time** | | **% at** | | | |  | **p** |
| --- | --- | --- | --- | --- | --- | --- | --- | --- |
|  | **Mean** | **Median** | **7^th^ day** | **8^th^ day** | **9^th^ day** | **Study end** |  |  |
| **Group I(Control)** | 7.0 | 7.0 | 0.0% | - | - | 0.0% | 12.422* | 0.002* |
| **Group II (Spiramycin treated)** | 7.83 | 8.0 | 66.7% | 16.7% | 0.0% | 0.0% |  |  |
| **Group III (*Penicillium gladioli* extract treated)** | 8.5 | 8.0 | 100.0% | 50.0% | 0.0% | 0.0% |  |  |
| **Pairwise comparison** | **P_1_= 0.019*, p_2_= 0.001*,p_3_ =0.001*** | | | | | |  |  |

χ^2^: Chi square for log rank test^.^ p_1_: p value for comparing between Control and Spiramycin treated, p_2_: p value for comparing between Control and *Penicillium gladioli* extract treated, p_3_: p value for comparing between Spiramycin and *Penicillium gladioli* extract treated. *: Statistically significant at p ≤ 0.05

**References**

1. Livak, K.J.; Schmittgen, T.D.J.m. Analysis of relative gene expression data using real-time quantitative PCR and the 2− ΔΔCT method. *Methods* **2001**, *25*, 402-408.
